# Exploring Drug Shortages in the United Kingdom

**DOI:** 10.3390/pharmacy11050166

**Published:** 2023-10-18

**Authors:** Mohamed Obiedalla, Nilesh Patel, Parastou Donyai

**Affiliations:** 1 Reading School of Pharmacy, University of Reading, Reading, Berkshire RG6 6AH, UK; nilesh.patel@reading.ac.uk; 2 School of Pharmacy, Kings College London, London WC2R 2LS, UK; parastou.donyai@kcl.ac.uk

**Keywords:** drug shortages, community pharmacists, patient care, COVID-19

## Abstract

Drugs can become short in supply for many reasons including increased demand and reduced production. Drug shortages have the potential to impact patients and pharmacists. This study aimed to highlight the challenges pharmacists face and the constraints of how they manage drug shortages. An online survey was designed with its link distributed electronically to community pharmacists in the UK with the assistance of pharmacy organizations during the period from September to December 2021. Survey data were analysed using descriptive statistics. A total of 83% of the respondents (*n*= 100) were experiencing drug shortages three or more times per week, and more than 70% of these spent 1–3 h per week dealing with them. A total of 93% of the respondents indicated that the issue of drug shortages was a problem for them, and 61% reported that it has worsened since the start of the pandemic. In addition, 65% of the respondents believed that drug shortages had had an impact on patient care. Drug shortages were shown to impact on the pharmacists’ workloads with a potential to affect the quality of patient care. There was a variation between how pharmacists dealt with drug shortages, which needs to be explored further with the reasons behind it.

## 1. Introduction

Pharmacists are considered the most accessible healthcare professionals due to the high volume of people who use their services on a regular basis. They are experts in medicines and use their professional knowledge and expertise to support patients with acute (immediate) and chronic (long term) conditions. The pharmacist profession worldwide has seen an increase in workload and mental health challenges since the start of the COVID-19 pandemic. Several studies have identified causative factors such as insufficient staffing levels due to sickness, increase in demand for pharmacy services and drug shortages [1,2,3,4,5]. In the United Kingdom (UK), even before the pandemic, pharmacists faced more demands on their time because of the need for them to provide more clinically oriented services that require additional accountability and responsibility by providing services such as minor ailments, smoking cessation, contraception and vaccination [6,7,8]. These pre-pandemic demands and the pandemic-related pressure have combined to create a crisis in the profession, certainly in the UK and potentially also worldwide.

The conventional route of medicine supply in the UK is via a prescription from the prescribing practitioner, which is presented to a pharmacy for dispensing. When there is a drug shortage, it is the community pharmacists who play a significant role in managing the shortages and getting the medicine to the patients. Pharmacists communicate with the patients, prescribers, wholesalers and manufacturers regarding the availability of drugs and suitable alternatives [9]. They also advise patients on the most suitable course of action to take when they are affected by the shortages.

Limited initiatives have been proposed to mitigate the issue of drug shortages. In the UK, the introduction of Serious Shortage Protocols (SSPs) has been one way of helping to reduce the impact of drug shortages. In February 2019, the Human Medicines Regulations (2012) were amended to introduce SSPs. In July 2019, further changes came into effect as part of the NHS (Amendments Relating to Serious Shortage Protocols) regulations 2019 [10]. SSPs provide community pharmacists the authority to amend or substitute a prescribed drug as set out in the protocol without having to refer back to the prescribing practitioner due to the drug being in short supply. Pharmacists can use their professional judgment in order to assess whether it is appropriate to supply a drug under the protocol. If the pharmacists have any concerns based on the history of the patient or individual circumstances, they can refer the patient back to the prescriber to have an alternative treatment. SSPs are not suitable for all drugs and patients. Furthermore, the use of SSPs requires the patient to consent to receiving the alternative treatment or the amendment. If the patient does not provide consent, they will be referred back to the prescriber for an alternative treatment.

Although there is not a global definition for drug shortages, the UK Department of Health and Social Care (DHSC) describes it as “a supply shortage of a presentation of health service medicine, [which] occurs when supply does not meet patient demand at national level” [11]. The issue of drug shortages is a global one, which has existed for a number of years [12] but was recently exacerbated by the COVID-19 pandemic [13,14,15]. The exact causes of these drug shortages, however, are multifactorial and not always known, but they include an increased demand and reduced supply [14,16,17,18,19]. For example, drugs such as hydroxychloroquine, midazolam and propofol were in short supply during the peak of the COVID-19 pandemic due to the increased use of them for the treatment of some COVID-19 cases [20]. Sudden changes in guidelines triggering a change in the prescribing practice can lead to increased demand. For example, in Canada, a shortage of beta blocker drugs was linked to a change in the management of hypertension guidelines [21]. In the UK, Brexit, which saw the withdrawal of the UK from the European Union, is believed to have impacted on supply, adding to the problem of drug shortages, possibly due to the fall in GBP and the trade barriers imposed as a result [22]. Regardless of the causes, drug shortages have the potential to impact on both patients and pharmacists at a personal and professional level.

Various studies have shown that drug shortages are experienced by both hospital and community pharmacists on a regular basis [23,24,25,26,27]. The Royal Pharmaceutical Society (RPS; professional body of pharmacists in the UK) also stated in 2022 that its members reported that the issue of drug shortages was a regular occurrence. In a Finnish study conducted in 2015, 129 community pharmacies were surveyed and reported to be experiencing drug shortages on a regular basis without apparent reasons [28]. The issue of drug shortages was also noted in a Polish study in 2020, which surveyed 435 pharmacists and found drug shortages to be a regular problem [29]. In a later bigger study in Serbia in 2021, which surveyed 500 public pharmacists, almost all pharmacists were experiencing drug shortages [30]. In surveys asking pharmacists about drug shortages, most respondents felt that drug shortages were on the increase and that they spent up to 6 h per week dealing with them [26,31,32]. From these studies, there appears to be some general consensus that pharmacists spend around an hour a day on average dealing with drug shortages. In a 10 h day, this could be estimated to take up to 10% of pharmacists’ work time, although this remains to be validated through observational studies. 

Despite the reported issues on workload, the impact of experiencing and dealing with drug shortages on pharmacists and patients has not been fully investigated, particularly within a community pharmacy where pharmacists experience them on a regular basis. Drug shortages can pose a threat to patient care by increasing the incidence of adverse events and medication errors or by delaying or interrupting treatment [33,34,35,36]. In a German study, which surveyed 482 community pharmacies, almost 90% of the respondents had dealt with drug shortages at least once in the past three months, which had an impact on patient care [37]. In addition to the clinical problems caused by drug shortages, there are also psychological implications for the patients such as worry, frustration and even anger towards the pharmacists for their inability to meet prescription demands [10,38]. 

With continuing problems of drug shortages, this study aimed to highlight the challenges community pharmacists face and the constraints in how they manage drug shortages. This includes potential changes in workload and an examination of barriers and facilitators to dealing with drug shortages. To achieve this, a survey was conducted to examine the experiences of pharmacists with drug shortages and how they deal with them, highlighting any inadequacies.

## 2. Materials and Methods

This was a cross-sectional study involving practising community pharmacists in the UK from September 2021 to December 2021 taking part in a self-administered online survey. In order to be eligible to participate, pharmacists were required to have been practising in the UK for at least 2 years. Pharmacists with less than 2 years of practice and retired pharmacists were excluded from the study. 

The initial pool of survey questions was developed based on anecdotal reports, published data on the impact of drug shortages on the work of pharmacists and the researchers’ own experience of community pharmacy practice. Thirty questions were initially drafted, and the response options were a mix of single answer or multiple answers. The survey was validated using content validity, which assessed whether the items in the survey were representative of the entire theoretical construct that the survey was designed to measure [39]. Content validity was performed by asking community pharmacists (*n* = 10) to provide feedback on each question based on their understanding, remembering, deciding on the answers and the relevance of the question. A Content Validity Index (CVI) was calculated for each of the items (question) using a scale (I-CVI). A minimum of 0.80 (I-CVI) was considered an acceptable value for an overall rating, which meant that 8 pharmacists out of 10 would give a rating of 3 or 4 (3 being agree and 4 being strongly agree) to the four CV questions (understand, remember, decide and relevant). Any item that did not meet the minimum I-CVI score of 0.8 was revised or removed. Following the results of the content validity analysis, the final version of the survey (Supplementary Materials Drug Shortage Survey version 2; DSS-v2) contained 29 questions. 

The survey (DSS-v2) contained two parts. The first part focused on drug shortages and included questions on prevalence, the impact of the pandemic, time spent dealing with drug shortages and communication about them. An open question allowed free-hand comments to be written on how to improve the management of drug shortages. The second part of the survey asked about the demographics of the pharmacists including their age group, level of experience, region of work and the type of work organization. 

The online survey included a Participant Information Sheet (PIS) and was made available as a link via the Online Survey platform. Participants were allowed access to the survey questions upon reading the PIS and their agreement to the consent statements. 

Purposive sampling was used, and the survey link was shared with the Pharmacists’ Defence Association (PDA), National Pharmacy Association (NPA) and Centre for Pharmacy Postgraduate Education (CPPE) for dissemination to their community pharmacist members. In addition, the social media platforms LinkedIn and Twitter were used to raise awareness of the survey. The survey was available for a period of three months after which no further responses were collected. 

Any identifiable data were processed as per the Data Protection Principles set out in the General Data Protection Regulations 2016 (GDPR) Act and the Data Protection Act 2018. The survey responses were checked to see if participants met the inclusion criteria (community pharmacists practicing in the UK for at least 2 years). No participants were excluded due to not meeting these criteria. Responses were then anonymised and transferred to an Excel spreadsheet and analysed using descriptive statistics. No correlations between the demographic data and the main responses were sought. Any free text comments provided were categorized and themed.

## 3. Results

A total of 100 responses were received from 11 regions in the UK. No data were missing from the analysis. All participants completed the questionnaire fully. Around three quarters of the respondents (73%, *n* = 73) had 5 years or more experience. Fifty-seven respondents worked in large multiples, twenty-three worked in small multiples and twenty worked in independent pharmacies (Table 1). 

All respondents had experienced drug shortages in the past and were experiencing drug shortages to some degree at the time of completing the survey. Eighty three percent of the respondents were experiencing drug shortages three or more times per week, and more than seventy % (*n* = 70) spent 1–3 h per week dealing with issues caused by drug shortages. 

The majority of the respondents (93%, *n* = 93) indicated that drug shortages were a problem for them, and this was spread evenly across the different types of pharmacy setting. 

**Figure 1:**
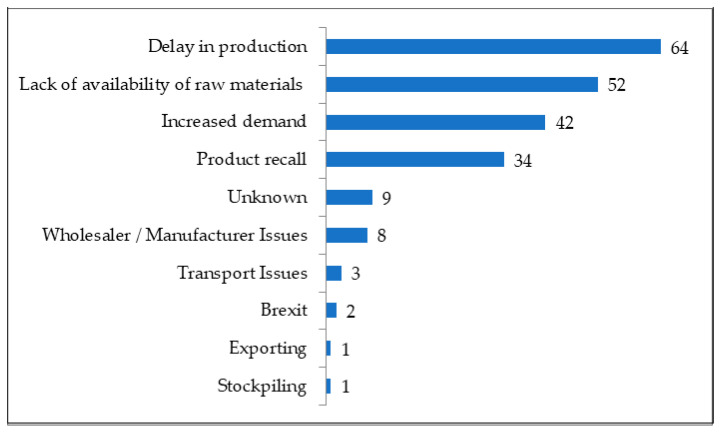
Reasons for drug shortages with frequency.

Increased demand, product recalls, delay in production and lack of availability of raw materials were all selected relatively evenly as the reasons for drug shortages by pharmacists (Figure 1). Additional comments included unknown reasons, wholesaler/manufacturer and transport issues, Brexit, exporting and stockpiling.

Sixty-one percent (*n* = 61) of respondents reported that the issue of drug shortages had worsened since the start of the pandemic.

Sixty-five percent (*n* = 65) of respondents believed that drug shortages had had an impact on them. Their comments were themed into groups, with most reporting the impact on patients to have been delays in their treatment (46/65). Other impacts were reported to be a loss of symptom control (8/65), increased anxiety and stress (8/65) and being hospitalised (3/65). More than half of the respondents (52%, *n* = 52) reported prioritising the supply and dispensing of drugs to more vulnerable/high risk patients based on the recent drug shortages.

The telephone was the most frequent method selected (75%, *n* = 75) for contacting other healthcare professionals to discuss drug shortages. Other methods selected included emails, WhatsApp or social media platforms and face to face meetings.

**Figure 2:**
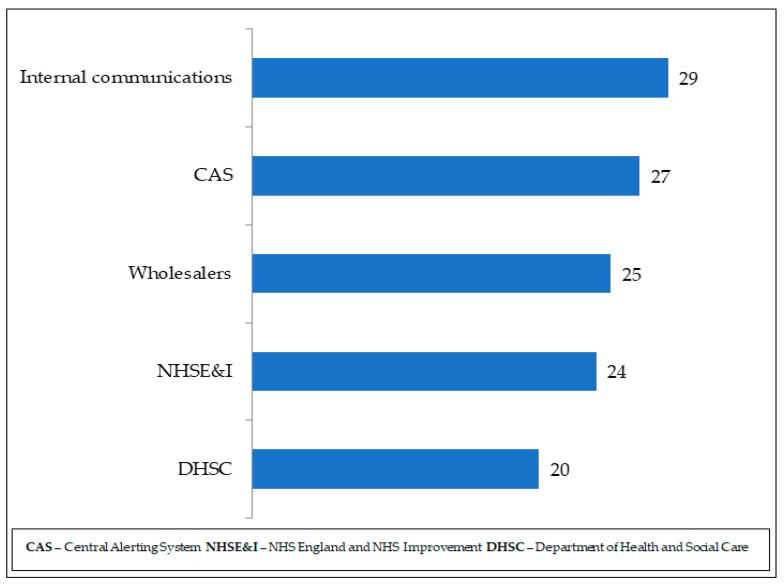
Drug shortages communication channels and frequency.

Sixty-one respondents (61%, *n* = 61) received drug shortage communications/alerts, whereas the others stated they either did not know or did not receive the communications. A range of sources informed pharmacists about drug shortages as shown in Figure 2, with internal communications being the most common source. The Pharmaceutical Services Negotiating Committee (PSNC), Welsh assembly government and Medicines and Healthcare products Regulatory Agency (MHRA) were listed as additional sources of communication about drug shortages. 

More than three quarters of the respondents (77%, *n* = 77) either did not know of or did not have a system such as a standard operating procedure (SOP) or protocol in their organizations that helped them to manage drug shortages.

**Figure 3:**
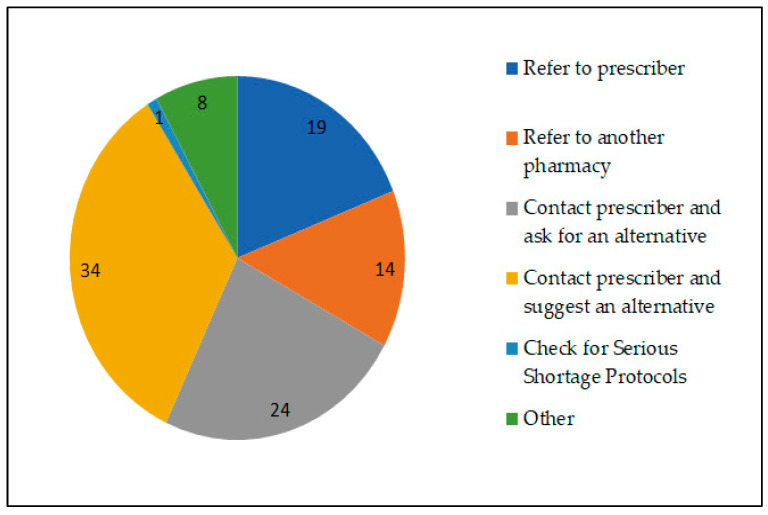
Method and frequency of dealing with prescriptions for drugs in short supply.

How the respondents dealt with prescriptions for drugs that were in shortage varied, with a third (34%, *n* = 34) responding that they would contact the prescriber and suggest an alternative (Figure 3). Additional comments indicated some (*n* = 4) would use any of the options provided depending on the circumstances, some (*n* = 2) would contact other pharmacies to ascertain stock levels and others (*n* = 2) stated they might ration the stock across multiple patients depending on the urgency. 

The respondents suggested the following for improving the management of drug shortages:

- Giving pharmacists more legal powers (*n* = 7).
- Increasing the number of Serious Shortage Protocols (SSPs) (*n* = 6).
- Providing early and transparent communications from the manufacturers or NHS with a list of alternatives and expected dates of resolution (*n* = 15).

## 4. Discussion

This study managed to highlight the challenges pharmacists faced and the constraints of how they managed drug shortages. The challenges were increased workload, lack of knowledge about drug shortages and inconsistency in the management of them. It was noted that no positive responses about drug shortages were reported. There was no difference between work organizations, although it might have been expected that large multiples with bigger buying power and storage facilities would experience drug shortages to a lesser extent than independent pharmacies and small multiples. Most of the respondents thought that drug shortages were a problem for them, experienced drug shortages three or more times a week and spent on average 1–3 h a week dealing with associated issues. This is in line with what has been reported by major pharmacy organizations that have also investigated this issue [27,32,33]. Furthermore, the prevalence of drug shortages in community pharmacies according to this study is similar to the Finnish, Polish and Serbian studies [29,30,31]. The time spent on dealing with drug shortages comes at a difficult time, particularly in the UK, when funding and contractual obligations are increasingly based on the provision of clinical services. To put this into context, 3 h a week can equate to twelve pharmacist consultations (15 min each), which can put a strain on pharmacists’ workloads, finances and potentially be detrimental to pharmacists’ wellbeing at work. Concerns regarding patient safety [34,36] as well as increased anxiety and stress for both patients and pharmacists as a consequence of drug shortages [32,38] have been reported. Furthermore, in this study, the majority of respondents believed that drug shortages had an impact on patient care. 

Most respondents believed drug shortages had worsened since the pandemic. The view that drug shortages have worsened since the pandemic is in line with that reported elsewhere [13,14,15]. These reports give a breadth of reasons for the problem, including consumer-related behaviours (e.g., panic buying) as well as supply related issues (e.g., unavailability of raw materials). Perhaps understandably, with the breadth of reasons for what might be causing drug shortages, it can be challenging to manage patient expectations about when the supply will be available in stock. Hence, transparent communications from the manufacturers or suppliers to the pharmacists would be very important in managing the expectations of patients.

The DHSC and NHSE&I developed a document in November 2019 detailing the national, regional and local management of communication and escalation processes of medicines supply and shortages. In our study, the majority of the respondents received communications to allow them to manage drug shortages in a timely manner. However, around one third of the respondents either did not receive or did not know about the communications, which may indicate there is more work that needs to be completed to improve this. As well as communications, having a clear process within pharmacy organizations to manage drug shortages may help to minimise the risks to patients who are affected.

As the rise in drug shortages continues, it is important for the pharmacy organizations to implement internal protocols in order to guide their pharmacists on how to deal with situations of drug shortages and help minimise the impact on their patients’ care. However, the survey data showed that two thirds of the respondents either did not have a drug shortage management system or did not know what the system was specifically. This is despite the RPS publishing a guide for dealing with medicine shortages in community pharmacies in March 2020 (a year before this study), which was recently updated. The guide provides tips and principles to help manage drug shortages effectively including sourcing or suggesting alternatives and having good communications between the pharmacy team, patients and other healthcare professionals. It is unclear if pharmacists in this study knew about or used this resource. Furthermore, access to RPS resources is usually available to RPS pharmacist members only, which the survey respondents may not have been.

There was a variation between how respondents indicated they dealt with drug shortages, with a third of the respondents stating that they would contact the prescriber to suggest an alternative way of dealing with the shortage. It might be reasonably expected that pharmacists contact the prescriber and suggest an alternative that is clinically suitable and available or check if there is an active SSP in place. However, in our study, only three of the respondents indicated that they would check for active SSPs, despite this being a legal framework for pharmacists to amend the drug on a prescription. The reasons for this are unclear; however, a report stated that pharmacists find SSPs complex and inflexible and did not know enough about them [10].

Despite the finding about the lack of use of SSPs, some respondents suggested that increasing the number of SSPs and giving pharmacists more legal powers to deal with drug shortages would be beneficial. Initiatives such as more legal powers have received attention by the RPS who believe that an amendment in law to allow pharmacists to make minor changes to prescriptions affected by drug shortages whilst using their clinical expertise and professional judgement would improve patient access to medicines and reduce the burden on the healthcare system [40]. It is unclear how this proposal would be implemented especially considering the low numbers of and use of SSPs.

Deciding the best way to ensure that the patient gets their medicine when there is a shortage is reliant on the pharmacists’ professional judgement, which in turn is based on other factors such as the drug that is in shortage, urgency of treatment, GP availability, clear communication about shortages and what to do about them, as well as the experience of the pharmacist. This could in turn mean a variation in the level of care received by the patients.

### 4.1. Strengths

This survey was content validated by community pharmacists and the final version was disseminated to UK community pharmacists. The respondents were from various pharmacy settings and had differing levels of experience. This study provides a baseline for the issues of drug shortages experienced by community pharmacists in the UK and highlights that further support is needed for dealing with drug shortages. 

### 4.2. Limitations

In light of the variation in the number of responses from the UK regions, it is difficult to undertake inferential statistics to fully verify these observations. Perhaps keeping the recruitment open for longer and using social media platforms may have increased the number of participants. We did not see any obvious bias from the non-responses to questions and do not think the method of recruitment would have introduced any bias that would have led to specific responses. The questionnaire was also content validated. However, the study may lack external validity due to the relatively low number of responses and the fact that some regions were over-represented, and others were under-represented. However, its aim of highlighting the challenges that pharmacists faced and the constraints on how they managed drug shortages was achieved. Given the fact that the RPS updated its guide for dealing with medicine shortages in community pharmacy in January 2023, perhaps carrying out this study after the update may result in different responses. The systems for managing drug shortages were not explored well enough as part of this study. Whilst we found an impact on workload when dealing with drug shortages, we did not determine whether specific drugs led to a variability in the workload commitment. This is something to investigate in a future study.

## 5. Conclusions

This study highlighted the challenges community pharmacists faced when dealing with drug shortages in the UK and the constraints with managing them. Drug shortages are an issue facing community pharmacists on a regular basis in the UK, which has worsened since the pandemic. Pharmacists seem to spend a considerable amount of time dealing with drug shortages in order to alleviate the impact on patient care. The reasons of drug shortages are not always clear to the pharmacists, which makes the management and addressing the patient expectations challenging. There is a need for more transparency and better communications between pharmaceutical companies/wholesalers and healthcare professionals. Giving pharmacists better support to resolve drug shortage situations for their patients may help improve patient access to treatment and reduce pharmacists’ time spent dealing with drug shortages. Of note, there were differences in how pharmacists dealt with drug shortages, which should be explored in future studies, for example by examining their behaviour towards dealing with drug shortage issues.

## Figures and Tables

**Table 1:** Demographic characteristics of community pharmacists.

Characteristic	Description	Number (*n* = 100)
Age	<25 years	2
	26–35 years	20
	36–45 years	28
	46–50 years	13
	51–60 years	27
	>60 year	10
Pharmacist experience	2–5 years	27
	6–10 years	15
	11–15 years	15
	16–20 years	8
	>20 years	35
Work location in UK	North East	4
	North West	19
	Yorkshire and the Humber	9
	West Midland	13
	East Midland	5
	South West	14
	South East	17
	East of England	7
	Northern Ireland	3
	Wales	1
	Scotland	8
Work organization	Independent pharmacy *	20
	Small multiples **	23
	Large multiples ***	57

* Less than 6 branches; ** between 6 and 99 branches; *** 100 or more branches.

## Data Availability

The data presented in this study are available on request from the corresponding author.

## Supplementary Materials

The following supporting information can be downloaded at: https://www.mdpi.com/article/10.3390/pharmacy11050166/s1.
