# Supplementary material for: Exploring Drug Shortages in the United Kingdom
Source: Pharmacy (Basel). 2023 Oct 18;11(5):166. doi: 10.3390/pharmacy11050166 (PMC10609979; doi:10.3390/pharmacy11050166)
Supplement: Supplementary file 1 [file pharmacy-11-00166-s001.zip › pharmacy-2553361-supplementary.pdf]

Drug Shortages Survey Questions

1. Are drug shortages a current problem for you?

- ◯ No
- ◯ Yes
- ◯ Don’t know

2. In your role, have you ever dealt with drug shortages?

- ◯ No
- ◯ Yes

3. In your role, have you ever dealt with patients affected by drug shortages?

- ◯ No (go to Q 16)
- ◯ Yes

4. On average, how many times a week do you experience drug shortages? (this is based on the number of times you have been unable to dispense or provide a prescribed or requested drug due to it being unavailable from the supplier / manufacturer)

- ◯ Once
- ◯ Twice
- ◯ Three
- ◯ More than three

5. On average per week, how long do you spend dealing with issues caused by drug shortages (hours)?

- ◯ None
- ◯ 1 - 3

○ 4 - 6

○ More than 6

6. Currently, is there a particular class or type of drugs you most commonly experience shortages with?

○ No

○ Yes (please specify below)

....

7. What have you found the reasons for drug shortages to be? (You may select more than one reason)

○ Increase in demand

○ Product recall

○ Delay in production

○ Lack of availability of raw materials

○ Other (Please specify below)

....

8. Based on your experience, what impact do you think Covid-19 has had on drug shortages?

○ Worsened drug shortages

○ Improved drug shortages (go to Q 10)

○ Has had no impact (go to Q 10)

○ Unsure (go to Q 10)

9. Is there any particular class or type of drugs that there has been a shortage of due to Covid-19?

○ No

○ Yes (please specify below)

....

10. Do you know if the drug shortage(s) you have dealt with recently within the last 12 months have had an impact on patient care?

- ○ No
- ○ Yes (please provide more information below)

....

11. Have you to prioritise the supply or dispensing of drugs to more vulnerable / high risk patients based on drug shortages in the past 12 months of practice?

- ○ No
- ○ Yes

12. Do you tend to communicate information to prescribers regarding drug shortages / alternatives prior to it affecting your patients' treatment?

- ○ No
- ○ Yes

13. How do you usually communicate information on drug shortages to prescribers or other healthcare professionals?

- ○ Via email
- ○ Via telephone
- ○ Via WhatsApp or other social media platform
- ○ Via face to face meetings
- ○ Other (please specify below)

....

14. Do you tend to communicate information to patients regarding drug shortages?

- ◯ No
- ◯ Yes

15. How do you usually deal with patients regarding drug shortages?

- ◯ Refer them to the prescriber
- ◯ Refer them to an alternative pharmacy
- ◯ Contact prescriber and ask for an alternative
- ◯ Contact prescriber and suggest an alternative
- ◯ See if there is an SSP
- ◯ Other (please specify below)

....

16. Do you receive drug shortage communications / Alerts?

- ◯ No (go to Q 18)
- ◯ Yes
- ◯ Don’t know (go to Q 18)

17. How do you usually receive alerts regarding drug shortages? (you may select more than one answer)

- ◯ Internal communications
- ◯ Wholesalers
- ◯ CAS (Central Alerting System)
- ◯ DHSC (Department of Health and Social Care) Medicines Supply Team
- ◯ NHSE&I (NHS England and NHS Improvement)
- ◯ Don’t know
- ◯ Other (please specify below)

…………………………………………………………………………………………………

18. Do you currently have a system in your organisation that manages drug shortages?

- No (go to Q 20)
- Yes
- Don’t know (go to Q 20)

19. Are you satisfied with the current system in place that manages drug shortages?

- No (please specify why below)
- Yes

……………………………………………………………………………………………..

20. Do you report new drug shortages?

- No
- Yes (please specify how below)

……………………………………………………………………………………………..

21. Which supplier do you use to source your drugs? (you may select more than one)

- Alliance Healthcare
- AAH
- Phoenix
- Direct manufacture purchase
- Other (please specify below)

……………………………………………………………………………………………..

22. Can you suggest improved ways of managing drug shortages?

....

Demographics

23. Age group (years):

- Under 25
- 25 - 35
- 36 - 45
- 46 – 50
- 51 - 60
- Over 60

24. Your work location:

- North East
- North West
- Yorkshire and the Humber
- West Midland
- East Midland
- South West
- South East
- East of England
- Northern Ireland
- Wales
- Scotland

25. Do you work in an?

- Independent pharmacy (less than 6 branches)
- Small multiples (between 6 and 99 branches)
- Large multiples (100 or more branches)

- ◯ Other (please specify below)

....

26. What is your current role / job title?

……………………………………………………………………………………………….

27. How long have you been working in your role (years)?

- ◯ Less than 1
- ◯ 1 - 3
- ◯ 4 - 6
- ◯ 7 - 9
- ◯ 10 or more

28. Do you work?

- ◯ Full time
- ◯ Part time
- ◯ As a locum

29. In the future, would you be interested in taking part in an interview to discuss your experiences of drug shortages?

- ◯ No
- ◯ Yes (please describe provide your email address below)

....

Your email address will only be accessible to the researchers, who will separate the email address from your survey responses before data analysis. Your email address will be saved in a password protected spreadsheet on secure university drives and will be deleted once the interview study is completed. You may contact the lead investigator at any point to ask for your email address to be deleted and not to be contacted again.
